# Project DECIDE II: evaluating the efficacy of supported advance care decision making within routine care in dementia: a randomized controlled trial

**DOI:** 10.1186/s12910-025-01290-6

**Published:** 2025-10-08

**Authors:** Ermioni Athanasiadi, Jochen René Thyrian, Janina Florack, Anna Theile-Schürholz, Jonas Karneboge, Tanja Müller, Kristian Kleinke, Stefanie Kremer, Charlotte Boes, Helene Böhm, Anja Herder-Peyrounette, Manuela Kremer, Celina Sander, Ronja Müller-Späth, Heiko Ullrich, Melanie Boekholt, Marc Hassenzahl, Ruben Albers, Simon Forstmeier, Philipp Schaper, Julia Haberstroh

**Affiliations:** 1 Department of Psychology, Faculty II, University of Siegen, Adolf-Reichwein-Str. 2a, Siegen, 57068 Germany; 2 Deutsches Zentrum für Neurodegenerative Erkrankungen (DZNE), Greifswald, Germany; 3 Alzheimer Gesellschaft Siegen-Wittgenstein e.V., Siegen, Germany; 4 Caritasverband Siegen-Wittgenstein e.V., Siegen, Germany; 5 Gesundheitsregion Siegerland, Siegen, Germany; 6 Klinikum Siegen, Siegen, Germany; 7 Ubiquitous Design, Faculty III, University of Siegen, Kohlbettstr. 15, Siegen, 57072 Germany

**Keywords:** Dementia care, Supported advance care decision-making, Proxy burden, Intervention

## Abstract

**Background:**

Dementia is a terminal illness and places significant burden on individuals and their caregivers, especially regarding end-of-life decisions for people with dementia (PwD) who lose their decision-making capacity. Current advance healthcare planning concepts fail to consider the psychological processes of decision making. Supported Advance Care Decision-Making (ACD) is a promising approach aiming to enrich existing advance healthcare planning concepts by integrating empowering support strategies and ensuring the success of empowerment. The study aims at providing a feasible and effective approach to enhance ACD in dementia care practice with the goal to increase PwD’s autonomy in making advance care and end-of-life decisions and to reduce proxy burden. It is embedded within Dementia Care Management (DeCM), a routine care measure specifically designed for PwD who live at home.

**Methods:**

The efficacy of supported ACD incorporated in DeCM routine care will be investigated with a single-blind randomized controlled trial. The primary endpoints are the prevalence and validity of advance healthcare planning documents (advance directives or other) and proxy burden at pre vs. post measurement. The secondary endpoints are decisional conflict, patient autonomy, and patient-proxy congruence in preferences.

**Discussion:**

Depending on the results, supported ACD shall be added to the curriculum of DeCM to further increase its effectiveness.

**Trial registration:**

DRKS00036478, 17.04.2025.

**Supplementary Information:**

The online version contains supplementary material available at 10.1186/s12910-025-01290-6.

## Background

Around 1,8 million people suffer from dementia in Germany, with an estimated increase of up to 2 million people within 10 years [1]. The German Hospice and Palliative Care Act (§132g SGB V, 1.12.2015) provides a framework for “Healthcare Planning for the Final Phase of Life”. Such advance healthcare planning^1^ is intended to ensure that individuals receive treatment and care as much as possible in accordance with their wishes, even if they are no longer able to give consent. In general, engaging in advance healthcare planning involves reflection and conversations about one’s own end of life. This is often a taboo topic, which can make it burdensome and anxiety-inducing for individuals and their families [2]. The absence of any form of advance healthcare planning ultimately results in the loss of patient autonomy. It also places a heavy burden on proxy^2^ decision-makers who face uncertainty regarding end-of-life decisions for people with dementia who have lost their decision-making capacity. This underscores the need for advance healthcare planning, with the goal to assist individuals in addressing this taboo topic to autonomously decide on an individually appropriate healthcare plan.

There are various approaches to advance healthcare planning such as advance care planning (often referred to as ACP), one of the most well-known concepts that in recent years has gained increasing relevance. A growing body of evidence demonstrates the effectiveness of advance care planning as a scientifically highly regarded approach to advance healthcare planning. Previous research has shown desirable effects on hospitalization, overall healthcare costs, patient and caregiver satisfaction, as well as on stress, anxiety, and depression in surviving relatives [3, 4]. While research on dementia-specific advance care planning has grown [5], there is still a lack of gerontopsychological research. Much of the effort to improve advance directive completion has focused on creating the perfect document and has overlooked the psychological process of decision-making and people’s wishes regarding the type of planning [6]. Advance care planning discussions align closely with standard practice; however, they often fall short of meeting the informed consent standard and, in many respects, are not suitable for individuals with dementia due to their complexity [7].

This study is part of the DECIDE-2^3^ project and aims to fill this gap by providing and evaluating measures to support the psychological process of decision-making in PwD. We refer to this concept of supported decision-making for advance decisions as *supported advance care decision-making (ACD)*. Conventional strategies for supported decision-making involve elements such as keyword lists and plain language. However, although they enjoy a high level of consensus, particularly among dementia researchers, clear evidence of their effectiveness has yet to be established [8], and experimental studies on supported decision-making, let alone on supported ACD, remain scarce. Within the first funding phase of the DECIDE project, we proposed a home-based, counselling-oriented approach to supported ACD which aims to improve advance healthcare planning for PwD [9]. The DECIDE-2 project builds on this work by offering and evaluating a tailored approach to support the psychological aspects of decision-making processes in advance healthcare planning within routine care.

### Objective

The goal is to increase the autonomy of PwD in making advance care and end-of-life decisions and to reduce proxy burden. We suggest an integrated approach for practical implementation into healthcare (see Study design section).

Our main research questions are:

1. Does supported ACD increase the prevalence and validity of advance healthcare planning documents for PwD living at home more than the gold standard of care?
2. Does supported ACD increase patient autonomy more than the gold standard of care?
3. Does supported ACD decrease proxy burden more than the gold standard of care?

We expect that the offer of supported ACD will lead to an increase in the prevalence and validity of advance healthcare planning documents, an increase in patient autonomy, and to a reduction of proxy burden.

This study is the first of two related subprojects of the jointly funded DECIDE-2 project. The overall purpose of this project is to improve advance healthcare planning in dementia care for both individuals with dementia and their proxy decision-makers. In both subprojects, integrated approaches suitable for practical implementation are investigated. In the first study, this involves incorporating Dementia Care Management (DeCM) as a routine-care concept and utilizing it as a vehicle for supported ACD.

The second subproject develops personalized chatbots to support early, easy-to-access ACD for older adults at risk of dementia (age $65+$) and proxies of PwD. Using co-design and iterative prototyping, it explores how conversational technology can support end-of-life planning at home. The goal is to help people reflect on their choices, explain them, and share them with loved ones. In a participatory process, the second subproject designs and evaluates conversational technology as a low-barrier, personalized, and ‘private’ way to engaging with ACD, aiming to provide support that facilitates access to additional conversations about the end of life in routine care. The second subproject is not part of this study protocol and will not be addressed further.

## Methods

### Participants

A total of $N = 150$ participants will be recruited in the period of March 2025 until January 2027 (see Required sample size and power analysis section for details on calculation and Recruitment section for recruitment details).

Inclusion criteria are a confirmed diagnosis of mild to moderate dementia (F00-F03) or mild cognitive disorder (F06.7), diagnosed by a physician specialised in neurology or psychiatry. The decision on whether to include a person with questionable capacity to consent will be based on a recommended procedure for involving non-consenting individuals in medical research [10]. Participants will be excluded from participation in cases of severe dementia (MMSE score $< 11$) and if lack of capacity to consent to study participation persists without an assisting proxy.

Additionally, every participating PwD will choose a trusted individual who will also participate in the study as proxy. If no such person is available, the PwD will still be considered for study participation.

### Intervention

**Fig. 1:**
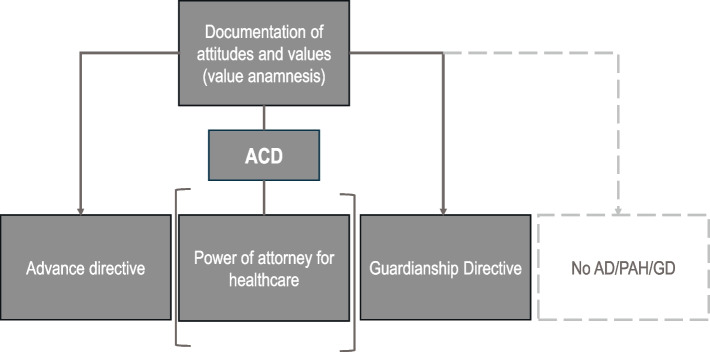
Integration of supported ACD into the process of advance healthcare planning [7]

The intervention lies in the *offer* (not mandatory participation) of supported ACD. Supported ACD is the process of supported decision-making and employs an integrated model that combines support (such as elaborated plain language, priority cards, and keyword lists) with capacity assessment. It aims to enable PwD to make self-determined decisions about their own healthcare planning. This involves deciding whether an advance care planning document should be created and, if so, which one. Advance healthcare planning can also mean that a person, after being informed about the various options for advance healthcare planning documents, makes a documented decision not to create such a document. Importantly, our intervention can be understood as an extension of advance healthcare planning (see Integration of supported ACD into advance healthcare planning section) and integrates the value anamnesis, a module of advance healthcare planning, as proposed by Voss [7, 11]. As part of the ACD, a value anamnesis as described in [7] is conducted first (see Fig. 1). This involves documenting general attitudes, values, and experiences related to living and dying, which are recorded in an interview. The next step involves the decision for or against the creation of advance healthcare documents. If a person decides to create advance healthcare documents, the following step is to determine which type of document should be prepared. The individual’s capacity to consent to this decision is documented, and the person with dementia is supported throughout the creation of the advance healthcare document.

### Integration of supported ACD into advance healthcare planning

Supported ACD strives to empower individuals with dementia to make self-determined decisions for or against *any* form of advance healthcare planning^4^. Importantly, the intervention extends the concept of advance healthcare planning, focusing on supported decision-making. As such, supported ACD can be viewed as an empowering module of advance healthcare planning which emphasizes the decision-making process rather than the planning. Aimed specifically to meet the needs of vulnerable individuals, its primary objective is to foster individuals’ capability for decision-making.

Conventional advance healthcare planning concepts lack information about alternative options for health-related advance planning and have recently faced criticism for disregarding the informed consent standard and for their complexity, which hampers their appropriateness for individuals with dementia [7]. Supported ACD aims to enrich advance healthcare planning concepts by integrating empowering support strategies and ensuring the success of this empowerment.

### Study design

This study is designed to compare supported ACD with the current gold standard in routine care for PwD. DeCM is an established routine care approach specifically developed for PwD living at home. We consider DeCM as the gold standard for routine care in Germany, as recently affirmed by its inclusion in the German S3-guideline for dementia. Though not routinely available yet in Germany, it is increasingly being implemented in conventional health care. In DeCM, a dementia care manager – a qualified nurse – visits the PwD at home and develops an individual care plan based on medical, nursing and psychosocial needs of the patient and their family, which are continuously assessed.

A parallel-group design will be used to study the effect of supported ACD within DeCM in a single-blind, randomized pre-post trial. Embedding supported ACD into DeCM allows the blinding of participants, as they will receive gold standard care in both study arms and no prior knowledge of the contents of DeCM is to be expected. No circumstances that require or allow unblinding were determined. Due to ethical reasons, participants in the control group will also receive the offer to receive supported ACD, but only after data collection is completed (to ensure that blinding is preserved). In this case, upon agreement, dementia care managers will again conduct supported ACD, but it will not be part of the study.

By integrating DeCM into both arms of the randomized controlled trial, the independent variable of the design is ACD, allowing us to attribute differences in the outcome to the offer of ACD. In this context, DeCM can be understood more as a vehicle that is well-suited to be complemented by supported ACD. We hereby go beyond a simple effect assessment towards a proof of effectiveness and clinical relevance.

### Randomization

Participants will be randomly assigned to a control (DeCM as routine care) or treatment (DeCM + supported ACD) arm. Randomization will be stratified by gender and study arm. Because previous research indicated increased dropout rates in the supported ACD condition [9], stratified randomization by study arm will be conducted in a 2:1 (treatment:control) ratio, which is expected to result in approximately balanced groups of complete data in the two studyarms. During data collection, the ratio will be monitored and adjusted as needed to ensure balanced groups by the end of the study.

The allocations will be computer-generated using *R* package blockrand [13] with a block size of 9 for studyarm allocation. Separate randomization lists will be kept by gender as well as for each dementia care manager to ensure balanced recruitment ratios across individuals.

### Procedure

#### Data collection

**Fig. 2:**
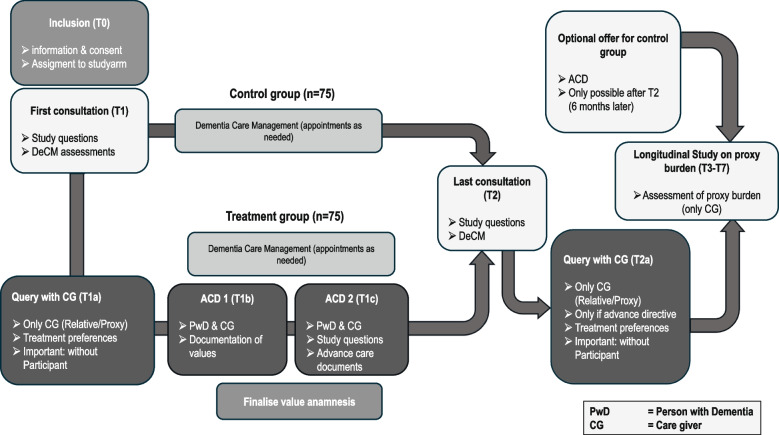
Design and scheduled appointments, assessments and interventions of the DECIDE-2 study

The procedure of the data collection process is shown in Fig. 2. Recruited individuals fulfilling the inclusion criteria will be asked to participate in the study. In the first appointment (T0), information about the study will be given and, if individuals wish to participate, consent for participation will be collected from the PwD as well as from their proxy. All information given to individuals prior to study inclusion will be standardized over both study arms and random assignment to study arm will take place afterwards.

In the first and the last DeCM consultation appointment (T1 and T2), participants in both study arms will answer relevant study questions containing the pre-post assessment of primary and secondary endpoints, as well as the assessment of DeCM (not part of the study, see Material section). The last DeCM consultation (T2) will be scheduled six months after T1 ($\pm$ 30 days). Participating proxies in the treatment arm will answer additional questions included in T1 (as $\text {T1}_{Treatment}$) concerning treatment preferences of the PwD. If an advance directive is present for participating PwD in the treatment arm at T2, their proxies will complete additional study questions in T2 concerning the PwD’s treatment preferences ($\text {T2}_{Treatment}$). In both of these cases, the participating PwD will be absent during this query, as this could influence the ACD process and relevant endpoints.

Between T1 and T2, participants in the treatment arm will complete two supported ACD appointments. The time window between baseline assessment (T1) and the first ACD appointment (ACD1) will be 1-30 days, and the time window between the two ACD appointments (ACD1 and ACD2) will be 1-15 days. In the first appointment (ACD1), participants will complete the value anamnesis as the first part of supported ACD with their proxy present. If required, it is possible to split the completion of the value anamnesis into several appointments to reduce stress for PwD. In the second ACD appointment (ACD2), participants in the treatment arm and their proxy will finalize the process of supported ACD, deciding on their preferred form of advance health care planning and receiving support in its implementation. If they choose an advance directive or a guardianship directive, the dementia care manager will assist in drafting the document. If they opt for a power of attorney, they will be advised to consult a notary or counselling centre for PwD. If they decide against any form of advance directive, this decision will be documented. In the case of a decision to create an advance directive, a standardized assessment of participants’ capacity to consent will be conducted. A document with the participants’ decision regarding their own advance healthcare planning will be handed over to individual participants and their proxies for further use.

Importantly, between T1 and T2, participants from both study arms will be able to receive routine care, i.e., attend, if needed, further home-based DeCM consultations offered by the dementia care managers. We suspect that the alleviating effects of the intervention on proxy burden may emerge increasingly in the long term. Therefore, for all participants who provide consent, follow-up assessments of proxy burden will be conducted after the end of the main study period over a period of five years using a longitudinal design (T3-T7). Participants who agree will be contacted annually via mail or email to complete the follow-up survey.

#### Recruitment

Recruitment will focus on patients or clients from the dementia care network in Siegen-Wittgenstein and will be further integrated within the locally operating DeCM (the dementia care managers will approach suitable patients in their work context). Recruitment will also extend to surrounding regions of Siegen-Wittgenstein to inform general practitioners, neurologists, pharmacies, associations, self-help groups, and the general public through local media channels. The network of participating practice partners represents the necessary care areas for DeCM and PwD (initial diagnosis, sector transitions, etc.) and includes an entire region with urban and rural areas. All partners have established care structures and prior experience in conducting and participating in scientific projects [14, 15], ensuring access to the target audience relevant to this trial. DeCM was already implemented in Siegen-Wittgenstein [16], and the dementia care managers already provide DeCM. They will invite patients or clients who meet the inclusion criteria. Based on our previous projects, recruitment in the hospital setting (geropsychiatry) will be supported by the physicians of the memory clinic located in the Klinikum Siegen.

#### Participant retention and withdrawal

To prevent selective drop-out due to cognitive impairments, several strategies will be applied to help participants attend their DeCM appointments. Upon agreement to participate, and if required, participants will receive reminder calls prior to the appointment. Missed appointments will be met with an offer to reschedule. If individuals decide to withdraw from participation, the reasons for dropping out of the study will be collected.

### Material

An overview of the instruments used for all measured variables is shown in Table 1. In addition to the existence of various advance health care documents (yes/no), the validity of an advance directive will be assessed, which is indicated by the existence of informed consent at the time the advance directive was created. For this purpose, a self-report questionnaire in accordance with the criteria for informed consent (adequate information, capacity to consent, voluntariness [17]) will be used.

**Table 1:** Overview of collected constructs and applied instruments

Measured variable	Instrument	Time	Target individual
Primary endpoints			
Presence and validity of advance care documents	closed questions based on DECIDE-I study [9], usage of advance directive	T1, T2, T3-T7	PwD and proxy
Proxy burden	Zarit Burden Interview (7 items, [18–21]), Burden Scale for Family Caregivers (BSFC-s, 10 items, [22]).	T1, T2, T3-T7	proxy
Secondary endpoints			
Patient autonomy	Decisional Autonomy Scale (DAS, [23]), Perceived Autonomy in Old Age scale (WAA, [24, 25])	T1, T2	PwD
Patient-proxy congruence with treatment preferences	two items with different versions for PwD and proxy, Statement of Treatment Preferences^a^ [26]	$\text {T1}_{Treatment}$+$\text {T2}_{Treatment}$(proxy), ACD2 (PwD)	PwD and proxy separately
Decisional Conflict	Decisional Conflict Scale (16 items, [27])	T1, T2	PwD
Moderators, Mediators, confounding variables			
Demographic variables	diagnosis, age, gender, education, employment, living situation	T1, T3-T7	PwD
Health-related preferences	health literacy (single item, see [28]), need for autonomy (single item, see [29]	T1	PwD
Health-related variables	single screening item & checklist based on Mini-DIPS (Short Structured Clinical Interview for diagnosing Mental Disorders) [30]	T1	PwD
Depression	Geriatric Depression Scale [31]	T1	PwD
Cognitive status	Mini Mental Status Test [32]	T1	PwD
Advance Care Decision-Making^a^			
Value anamnesis	According to [11]	ACD1	PwD
Capacity to consent	CAT-AD [33]	ACD2	PwD
Advance care documents	Template by Bavarian State Ministry	ACD2	PwD and proxy

T1-T7 are illustrated in detail in Fig. 2

^a^Only for participants in treatment arm who create an advance directive

Patient-proxy congruence is used to evaluate how accurately the future proxy can assess the treatment preferences of the PwD. It will be assessed with two items developed for this purpose, both of which will be answered by the future proxy and by the PwD. For participants in the treatment arm who create an advance directive, the statement of treatment preferences will also be collected, for which three scenarios from [34] were adapted according to the target population. Details are found in Additional file 1. While the hypothetical scenarios will be presented directly to the proxy, PwD will be exposed indirectly – in the context of the advance directive process – to the scenarios in order to minimize burden for the individual.

The implementation of ACD is divided into the collection of the value anamnesis [11], the examination of the capacity to consent using the CAT-AD [33], and the optional creation of advance documents. The CAT-AD is an adaptation of the MacCAT-T [35, 36] and DCAT-PAD [37, 38]. It has already been applied and validated in the DECIDE project, achieving an interrater reliability of $> 80\%$ per item, while the agreement with the overall scale of capacity to consent was 100% [33]. While the CAT-AD is evaluated for study purposes as part of supervision and quality control, the value anamnesis and the advance documents will be left with the participants for personal use and will not be further used for study purposes.

To simplify the data collection process for the cognitively impaired target group, the rating scale was uniformly adapted to a 5-point Likert scale (“strongly disagree” to “strongly agree”) for the majority of the questionnaires and the wording of the questions was adapted accordingly. Lastly, DeCM will be assessed with a range of questions developed and evaluated in a past study focusing on the implementation of DeCM [16]. These assessments do not represent an endpoint in the present study and are not further described in this protocol (for more details, see [16]).

### Outcome measures

The primary and secondary endpoints were chosen based on DeCM [39, 40] and advance care planning trials [4, 5]. The primary endpoints are the prevalence and validity of advance healthcare planning documents (advance directives or others) and proxy burden at pre- vs. post-measurement. Secondary endpoints are decisional conflict, patient autonomy, and patient-proxy congruence in preference. Details about the measurement of endpoints are provided in Material section.

Furthermore, the following demographic and clinical variables will be recorded: age, gender, education level, psychiatric comorbidities, health literacy, and the need for autonomy in medical decision-making. Although only age has previously been shown to be associated with the primary endpoints [41], the additional variables will be collected to provide a comprehensive description of the study population and to enable exploratory analyses.

### Statistical methods

#### Required sample size and power analysis

The use of advance directives remains insufficiently widespread in Germany. Based on a German sample of 4,185 individuals between 50 and 90 years [42] and previous research with the target population [41], we anticipate a baseline prevalence of approximately 50% in our study population. Furthermore, the validity of existing advance directives is questionable in about 50% of cases [43]. The planned intervention will be considered practically relevant if this frequency increases by at least about 20-30 percentage points. Sample size calculations and simulations using the R package mixedpower showed that about 150 participants will be necessary to detect effects in the moderate range with sufficient power ($> .8$) in the focal effect of interest: i.e. the condition-by- time interaction in the (generalized) linear mixed-effects models (described in Statistical analyses section) both regarding our primary and secondary endpoints (see Outcome measures section).

#### Statistical analyses

Assessment of the prevalence and validity of advance directives will be based on Bayesian generalized linear mixed-effects models. Bayesian estimation is more appropriate than maximum likelihood for smaller sample sizes, when the priors are chosen carefully [44]. We will use probit models modeling the change in the probability of the presence and validity of advance directives over time given the experimental condition and possible covariates (see Outcome measures section).

Outcome variables in these models are a) the binary variable for presence of an advance directive (yes/no), and b) the binary variable for the validity of the advance directive in the sense that it was both voluntarily given and based on informed consent (yes/no). In these models, the condition-by-time interaction and the contrast (whether the pre-post change in the ACD + DeCM condition is more pronounced than in the DeCM condition) are of primary interest. Likewise, analyses of proxy burden and of secondary endpoints will be based on Bayesian linear mixed effects models. Scale scores based on 5-point Likert scale items usually justify the use of ‘normal’ linear mixed effects models. If this is not feasible, the outcomes will be modeled as (ordered) categorical. To avoid loss of sample size, and consequently loss of statistical power, missing data in all model variables will be multiply imputed where appropriate (see Exploratory statistical solution development for missing data and small sample sizes section).

### Methodological side projects

#### Exploratory statistical solution development for missing data and small sample sizes

Missing values in randomized controlled trials commonly pose challenges for accurate statistical inference, especially when sample sizes are (very) small, which is commonly the case in dementia research. The emerging challenges, such as loss of power and model identification issues, can be addressed through methods such as multiple imputation (MI). Previous research found that MI under *Missing At Random* (MAR) could yield acceptable inferences even for small-sample-sizes [45, 46], and could outperform other missing data handling techniques in small sample scenarios [47].

However, the particular approach that is being used heavily influences the obtained results, and adopting the default methods and settings of standard MI software might not be the best option available [48, 49]. Until now, there is little research regarding these consequences in the binary outcome context, where the required sample size further depends on the imbalance of event rates in the outcome. While MI has been found to be superior to other methods in the context of logistic regression [50], the question remains whether a logistic MI model is still feasible when sample size is very small.

Using Monte Carlo simulations, this subproject aims to investigate the performance of logit and probit MI models to handle missing values in small sample size scenarios. Looking beyond common MI solutions, we will also explore fully Bayesian analytical approaches as well as maximum likelihood approaches. The overall aim is to provide practical guidelines regarding how to properly treat missing data in small sample size scenarios and to apply them to missing value handling in the main study.

#### Interrater reliability of Capacity-to-Consent-Judgments of dementia care managers vs. psychotherapists

In the current study, dementia care managers will assess the capacity to consent for (or against) the creation of an advance directive. The extent to which dementia care managers align with physicians and psychotherapists in assessing the capacity to consent (interrater reliability) will be examined in this side project with two sub-studies.

In the first sub-study of the interrater side project, the conversations conducted by dementia care managers for the assessment of capacity to consent will be recorded if agreed to by the PwD; a physician or psychotherapist will then provide a second judgment on capacity to consent based on the recordings. The participating physicians and psychotherapists are recognized experts in capacity assessment.

The second sub-study of the interrater side project will assess inter-rater agreement between nursing professionals and physicians or psychotherapists in an online study. Similar to the study of Haberstroh & Müller [51], it will utilize a case vignette of an individual with mild dementia. The presentation and structure of the case will follow the guideline for assessing capacity to consent, developed in collaboration with the American Bar Association and the American Psychological Association [52]. In this online study, participants will first be presented with the criteria for capacity to consent, then they will be shown a case vignette and asked to assess the patient’s capacity to consent based on the vignette.

### Quality control

For quality assurance and control, all study personnel will receive training in content-related, methodological, ethical, legal, and organizational aspects [53]. All dementia care managers have undergone comprehensive ACD-training provided by experts, equipping them with specific knowledge and skills required to conduct supported ACD with the target population. A small pilot study ($n < 5$) will be conducted and evaluated prior to the main study so that any required modifications can be incorporated into the manual of operations. Necessary changes to established procedures will be justified, documented and communicated to all study personnel in a timely manner. Any amendment will be submitted to the responsible ethics committee.

#### Data management

##### **Data collection**

Data collection conducted by the dementia care managers will be computer-assisted using the proprietary software of the German Center for Neurodegenerative Diseases in Greifswald and will be stored on the Center’s local servers. For the subpopulation of participants who create an advance directive as part of the study, audio recordings of the approximately 20-minute interview section to assess capacity to consent (CAT-AD) will be made using dedicated digital voice recorders. These recordings will be re-evaluated by the psychotherapists from the research team to assess inter-rater reliability and to ensure quality control of the assessment.

All personnel managing personal data as part of the research project will be made aware of applicable legal requirements and potential risks. Before the start of fieldwork, those involved in data collection will be thoroughly prepared, trained and their activities will be constantly monitored. Data collection will be accompanied by continuous data monitoring and standardized reporting. Monitoring will focus on aspects like missing values, implausible values, and value distributions. The study has been registered in the German Clinical Trials Register (DRKS Trial no. DRKS00036478) and this protocol was written following the SPIRIT guidelines (see Additional file 2). External quality will be further assured through external expert advice on ethics (see Ethical considerations section).

##### **Confidentiality, data access and storage**

Personal data will be anonymized before being entered into the project database and all forms of data processing will be conducted in compliance with the EU General Data Protection Regulation (GDPR) and applicable national data protection laws, ensuring data protection. Project data will be retained at the principal investigators’ institutes for at least 10 years after the funding period ends. Anonymized research data will be archived for long-term preservation (e.g., via the research data center at the Leibniz Institute for Psychology) and (as far as possible) made accessible to interested parties upon reasonable request.

The audio recordings will be stored in the university cloud, password-protected, and deleted after evaluation. They will only be accessible to a limited group of individuals (responsible dementia care manager, study physician/psychotherapist). In the case of conflicting judgments, the audio file will be given to a third qualified decision-assistance person (study physician or psychotherapist) for independent assessment.

### Ethical considerations

The study has been reviewed and approved by the Ethics Committee of the Medical Council Westfalen-Lippe Council Westfalen-Lippe (trial no. 2024-767-f-S). A list containing the items of the WHO Trial Registration Data Set was completed (see Additional file 3). In case of any relevant changes to this protocol, both the study registry and the ethics committees will be informed and asked for approval.

#### Informed consent

The capability of providing informed consent can be an ethical issue in individuals with dementia. In accordance with the German AWMF (Association of Scientific Medical Societies in Germany) S2k guidelines for medical procedures (“Einwilligung von Menschen mit Demenz in medizinische Maßnahmen” [17]), participants will be supported as far as possible (e.g. through use of keyword lists, plain language). In questionable cases, consent by proxy will be considered, but the participant’s assent will still be required. In such cases, the decision on whether to include a person with questionable capacity to consent will be based on a recommended procedure for including non-consenting individuals in medical research [10].

Following European Commission Guidelines, we will carefully design informed consent forms and processes as well as information sheets based on the results of our previous research [54, 55] and on already existing documents of the RoutineDeCM project [16].

#### Possible harm

PwD in the intervention group will receive two additional appointments including decision-making situations, which can be stress-inducing for the PwD. To minimize adverse events and negative psychological symptoms, tailored support strategies will be used. To avoid overload, the process of creation of advance care documents will be spread over two appointments. Other than this, no possible harm or adverse events are expected. Treatment and care will follow current guidelines and be supervised and coordinated by clinical experts. The dementia care managers are specifically trained and qualified for the tasks involved in conducting the intervention. This includes an emergency plan, phone availability of supervisors during household visits as well as intense training by psychotherapists to handle adverse events. Case conferences with clinical supervisors will be conducted on a regular basis, enabling the dementia care managers to discuss and receive consultation for experiences made in the household.

#### Public dissemination, transfer and implementation

The obtained scientific findings will be submitted to peer-reviewed, preferably open-access journals for publication and presented at scientific conferences and meetings, including the annual network meeting of all stakeholders engaged in the National Dementia Strategy. No hiring of professional writers is intended. The results will be further shared with the broader public through regional workshops, online articles, and public talks, to improve dissemination beyond the scientific community.

Guidance on questions related to research ethics will be provided by an independent ethics advisory board from the Institute for Medical Ethics and History of Medicine at Ruhr-University Bochum. Additionally, our patient advisory board as well as our advisory board for relatives, both established with the Alzheimer Society Siegen, will be actively engaged in the process of public dissemination, transfer and implementation. The boards will offer practical and ethical advice, ensuring the research aligns with the needs of PwD and their relatives. The collaborative nature of participatory research inherently fosters a sense of ownership among stakeholders, significantly enhancing the probability of successful implementation.

## Discussion

Through its implementation-based nature, this trial aims to contribute both to clinical practice and to the broader field of dementia research. The findings shall provide valuable insights into the feasibility and effectiveness of supported ACD in routine dementia care. If shown to be beneficial, the proposed intervention could offer a structured approach to integrating ACD into healthcare services. By embedding the intervention within clinical practice, the study seeks to generate findings that are not only theoretically relevant but also practically applicable for stakeholders in the healthcare system.

A notable strength of this study is its single-blind design, which is particularly challenging to achieve in psychosocial research due to the nature of the intervention and the active involvement of participants. This approach minimizes potential biases and enhances the study’s internal validity. However, complete blinding is not feasible, as DeCMs are part of the research team and the additional ACD appointments represent a visible modification to routine care.

Furthermore, the study follows a participatory approach by actively engaging relevant stakeholders from healthcare practice - including dementia care managers, clinicians, PwD and family caregivers - throughout the design and implementation phases. This collaborative involvement aims to enhance the relevance and feasibility of the intervention by ensuring that it aligns with the needs of both the target population and those responsible for its delivery.

Supported ACD may serve as a valuable addition to existing advance healthcare planning approaches, particularly for PwD. It has the potential to enhance informed decision-making by providing structured decision support and standardized assessments of decision-making capacity. Future research should examine whether supported ACD could complement standard advance healthcare planning processes beyond dementia care, contributing to the overall validity and transparency of advance healthcare planning.

Despite its strengths, this study also presents several challenges. One key issue is the potential for selective sampling. More severely impaired individuals may be more motivated to participate in the study due to an already heightened awareness of the burdens associated with dementia, possibly leading to a non-representative sample. To ensure voluntary participation, individuals who opt out of the study will continue to receive DeCM as part of routine care. Another challenge is participant retention, given the progressive nature of dementia. Symptom progression may contribute to increased attrition rates and selective drop-out, potentially reducing the sample size and leading to missing-not-at-random (MNAR) data patterns. These challenges will be addressed through an adjusted sampling strategy during recruitment as well as through appropriate statistical methods and insights from related subprojects focusing on missing data. Furthermore, in our study, dementia care managers, rather than physicians or psychotherapists (which is the conventional practice) will assess participants’ capacity to consent. To ensure the validity of the judgments, they will be reviewed by physicians or psychotherapists with expertise in the field in a subsequent sub-study. Finally, the generalizability of the findings may be limited by the study’s regional scope. The results could be influenced by local healthcare policies and care structures, necessitating further research to explore how supported ACD might be adapted to different healthcare settings and geographic contexts.

In summary, this study aims to contribute empirical evidence on the role of supported ACD in dementia care. The findings will help assess whether routine care for PwD can be enhanced through structured decision support and may inform discussions on the broader applicability of ACD in healthcare. Future research should explore the transferability of these findings to different healthcare systems and investigate long-term outcomes associated with the integration of ACD into routine practice.

## Supplementary Information

Supplementary Material 1.

Supplementary Material 2.

Supplementary Material 3.

## Data Availability

No datasets were generated or analysed during the current study.

## Abbreviations

PwD: People with dementia
ACD: Advance care decision-making
DeCM: Dementia care management
AWMF: Association of scientific medical societies in Germany
MI: Multiple imputation
MAR: Missing at random

## Footnotes

1 We understand advance healthcare planning as a general term that refers to all possible forms of planning ahead for medical care, especially advance directives, powers of attorney, guardianship, guardianship directives and Advance Care Planning.

2 The term *proxy* refers to a trusted person who is designated to make healthcare decisions on behalf of a PwD. A proxy may, but does not have to be, a caregiver. In the context of advance healthcare planning, proxies are specifically named in an advance directive. In this protocol, the term *proxy* may refer to a current, potential, or future decision-maker.

3 Funding number: 01GY2405

4 The term *advance healthcare planning* or *advance care planning* generally describes *“a process that supports adults at any age or stage of health in understanding and sharing their personal values, life goals, and preferences regarding future medical care. The goal of advance care planning is to help ensure that people receive medical care that is consistent with their values, goals and preferences during serious and chronic illness. For many people, this process may include choosing and preparing another trusted person or persons to make medical decisions in the event the person can no longer make his or her own decisions.”* [12]. In the international context, it refers broadly to a range of practices and strategies aimed at supporting patient autonomy. In Germany, “advance care planning” or its abbreviation *ACP* is often used to describe a specific program of advance healthcare planning. In our work, we understand the term “advance healthcare planning” in its broader, generic sense and do not use it synonymously with any particular type of program or protocol.
