# Supplementary material for: Project DECIDE II: evaluating the efficacy of supported advance care decision making within routine care in dementia: a randomized controlled trial
Source: BMC Med Ethics. 2025 Oct 8;26:124. doi: 10.1186/s12910-025-01290-6 (PMC12505856; doi:10.1186/s12910-025-01290-6)
Supplement: Supplementary file 1 — Supplementary Material 1. [file 12910_2025_1290_MOESM1_ESM.pdf]

Patient-Proxy Congruence (MmD)

1. Wie sehr trifft diese Aussage auf Sie zu: „Ich bin mir sicher, dass meine Vertrauensperson meine Präferenzen und Wünsche in Bezug auf medizinische Entscheidungen am Lebensende kennt."

- ☐ Trifft überhaupt nicht zu
- ☐ Trifft eher nicht zu
- ☐ Trifft teils / teils zu
- ☐ Trifft eher zu
- ☐ Trifft genau zu
- ☐ Weiß nicht
- ☐ Antwortverweigerung

2. Wie sehr trifft die diese Aussage auf Sie zu: „Ich bin mir sicher, dass meine Vertrauensperson bei medizinischen Entscheidungen am Lebensende meine Präferenzen und Wünschen stellvertreten kann."

- ☐ Trifft überhaupt nicht zu
- ☐ Trifft eher nicht zu
- ☐ Trifft teils / teils zu
- ☐ Trifft eher zu
- ☐ Trifft genau zu
- ☐ Weiß nicht
- ☐ Antwortverweigerung

Patient-Proxy Congruence (VP)

3. Wie sehr trifft diese Aussage auf Sie zu: „Ich bin mir sicher, die Präferenzen und Wünsche meines Angehörigen in Bezug auf medizinische Entscheidungen am Lebensende zu kennen.

- ☐ Trifft überhaupt nicht zu
- ☐ Trifft eher nicht zu
- ☐ Trifft teils / teils zu
- ☐ Trifft eher zu
- ☐ Trifft genau zu
- ☐ Weiß nicht
- ☐ Antwortverweigerung

4. Wie sehr trifft diese Aussage auf Sie zu: „Ich bin mir sicher, dass ich bei medizinischen Entscheidungen am Lebensende die Präferenzen und Wünsche meines Angehörigen stellvertreten kann.“

   ☐ Trifft überhaupt nicht zu

   ☐ Trifft eher nicht zu

   ☐ Trifft teils / teils zu

   ☐ Trifft eher zu

   ☐ Trifft genau zu

   ☐ Weiß nicht

   ☐ Antwortverweigerung

Statement of Treatment Preferences (VP)

Einleitung:
Patientenverfügungen gelten für den Fall, dass eine Person nicht mehr selbst entscheiden kann. Dieser Fall kann z.B. bei Bewusstlosigkeit oder weit fortgeschrittener Demenz auftreten. Bei Einwilligungsunfähigkeit ist es die Aufgabe des Betreuers oder Vorsorgebevollmächtigten eine Entscheidung zu treffen, die den Wünschen und Vorstellungen des Betroffenen entspricht. Patientenverfügungen sind in diesem Fall eine wichtige Hilfestellung.

Wir möchten Sie bitten für die nachfolgenden Fallbeispiele zu entscheiden, was aus Ihrer Sicht am ehesten den Wünschen und/oder dem mutmaßlichen Willen Ihres Angehörigen entspricht. Sie können für jede Situation ein Behandlungsziel wählen: Entweder Lebensverlängerung oder Leidenslinderung.

Situation 1: Bleibende Einwilligungs- und Entscheidungsunfähigkeit

5. Stellen Sie sich vor, [Name Angehörige/r] erleidet jetzt einen schweren Unfall, einen Schlaganfall oder einen Herzinfarkt. Nach den ersten Notfallmaßnahmen und einer sorgfältigen ärztlichen Untersuchung halten es die Ärzte für sehr unwahrscheinlich, dass er seine/sie ihre geistigen Fähigkeiten wiedererlangen wird.

   In einer solchen Situation würde [Name Angehörige/r] wünschen:

☐ Lebensverlängerung: d.h. uneingeschränkte Durchführung von Maßnahmen, die das Leben verlängern (Behandlung auf einer Intensivstation, künstliche Ernährung)

☐ Leidenslinderung: d.h. Verzicht auf Maßnahmen, die das Leben verlängern würden (z.B. künstliche Ernährung) und stattdessen ausschließlich leidenslindernde Maßnahmen

Situation 2: Behandlung von unerträglichen Schmerzen und anderen belastenden Symptomen bei tödlich verlaufender Erkrankung

6. Stellen Sie sich vor, [Name Angehörige/r] leidet im Endstadium einer tödlich verlaufenden Erkrankung (z.B. Demenz, Krebserkrankung) unter unerträglichen Schmerzen und/oder anderen belastenden Symptomen wie z.B. Atemnot, Angst oder Übelkeit.

Wie möchte [Name Angehörige/r] bei in diesem Fall behandelt werden:

☐ Lebensverlängerung: d.h. Lebensverlängerung und Wachheit sind für [Name Angehörige/r] wichtiger als die umfassende Behandlung von Schmerzen.

☐ Leidenslinderung: d.h. [Name Angehörige/r] wünscht eine umfassende Behandlung von Schmerzen und nimmt dafür in Kauf, dass sein/ihr Bewusstsein möglicherweise getrübt wird. Auch die verbleibende Lebenszeit kann kürzer sein

Situation 3: Notfall

7. Stellen Sie sich vor, [Name Angehörige/r] wird im Endstadium einer tödlich verlaufenden Erkrankung (z.B. Demenz, Krebserkrankung) stationär in einem Krankenhaus behandelt und erleidet dort einen Herz- und/oder Atemstillstand.

   In dieser Situation wünscht [Name Angehörige/r]

☐ Lebensverlängerung: d.h. wiederbelebt zu werden (akzeptieren einer Herz-Lungen-Wiederbelebung).

☐ Leidenslinderung: d.h. nicht wiederbelebt zu werden (und das Sterben zuzulassen)
