# Supplementary material for: Project DECIDE II: evaluating the efficacy of supported advance care decision making within routine care in dementia: a randomized controlled trial
Source: BMC Med Ethics. 2025 Oct 8;26:124. doi: 10.1186/s12910-025-01290-6 (PMC12505856; doi:10.1186/s12910-025-01290-6)
Supplement: Supplementary file 2 — Supplementary Material 2. [file 12910_2025_1290_MOESM2_ESM.pdf]

Standard Protocol Items: Recommendations for Interventional Trials

SPIRIT 2013 Checklist: Recommended items to address in a clinical trial protocol and related documents*

Section/item	Item No	Description
**Administrative information**		
Title	1	Descriptive title identifying the study design, population, interventions, and, if applicable, trial acronym<br>*see Title Page*
Trial registration	2a	Trial identifier and registry name. If not yet registered, name of intended registry<br>*between Abstract and Keywords*
	2b	All items from the World Health Organization Trial Registration Data Set<br>*see Appendix*
Protocol version	3	Date and version identifier<br>*following the title page*
Funding	4	Sources and types of financial, material, and other support<br>*see section Declarations, subheading Funding*
Roles and responsibilities	5a	Names, affiliations, and roles of protocol contributors<br>*see Title Page and section Declarations, subheading Author's Contributions*
	5b	Name and contact information for the trial sponsor<br>*following the Title Page*
	5c	Role of study sponsor and funders, if any, in study design; collection, management, analysis, and interpretation of data; writing of the report; and the decision to submit the report for publication, including whether they will have ultimate authority over any of these activities<br>*see section Declarations, subheading Funding*
	5d	Composition, roles, and responsibilities of the coordinating centre, steering committee, endpoint adjudication committee, data management team, and other individuals or groups overseeing the trial, if applicable (see Item 21a for data monitoring committee)<br>*see section Quality Control, subheading Data Management: Confidentiality, Data Access and Storage*

Introduction

Background and rationale	6a	Description of research question and justification for undertaking the trial, including summary of relevant studies (published and unpublished) examining benefits and harms for each intervention<br>*See section Background*
	6b	Explanation for choice of comparators<br>*See section Study Design for the description of the parallel-group design*
Objectives	7	Specific objectives or hypotheses<br>*See section Background, subheading Objective*
Trial design	8	Description of trial design including type of trial (eg, parallel group, crossover, factorial, single group), allocation ratio, and framework (eg, superiority, equivalence, noninferiority, exploratory)<br>*Trial design: See sections Study Design and Intervention*<br>*Framework: See sections Study Design and Distinction between supported ACD and ACP*

Methods: Participants, interventions, and outcomes

Study setting	9	Description of study settings (eg, community clinic, academic hospital) and list of countries where data will be collected. Reference to where list of study sites can be obtained<br>*Description of study settings and list of study sites: see section Methods, subheading Participants and section Procedure, subheading Recruitment*<br>*countries of data collection: n/a*
Eligibility criteria	10	Inclusion and exclusion criteria for participants. If applicable, eligibility criteria for study centres and individuals who will perform the interventions (eg, surgeons, psychotherapists)<br>*see section Participants, second paragraph*
Interventions	11a	Interventions for each group with sufficient detail to allow replication, including how and when they will be administered<br>*see sections Intervention, and section Procedure subsection data collection*
	11b	Criteria for discontinuing or modifying allocated interventions for a given trial participant (eg, drug dose change in response to harms, participant request, or improving/worsening disease)<br>*n/a*
	11c	Strategies to improve adherence to intervention protocols, and any procedures for monitoring adherence (eg, drug tablet return, laboratory tests)<br>*see section Procedure, subheading Participant retention and withdrawal*

	11d	Relevant concomitant care and interventions that are permitted or prohibited during the trial<br>*see section Participants, second paragraph, last sentence*
Outcomes	12	Primary, secondary, and other outcomes, including the specific measurement variable (eg, systolic blood pressure), analysis metric (eg, change from baseline, final value, time to event), method of aggregation (eg, median, proportion), and time point for each outcome. Explanation of the clinical relevance of chosen efficacy and harm outcomes is strongly recommended<br>*See section Outcomes and table 1*
Participant timeline	13	Time schedule of enrolment, interventions (including any run-ins and washouts), assessments, and visits for participants. A schematic diagram is highly recommended (see Figure)<br>*see section Procedure subsection data collection (including Figure 1)*
Sample size	14	Estimated number of participants needed to achieve study objectives and how it was determined, including clinical and statistical assumptions supporting any sample size calculations<br>*see section Statistical Methods subheading Required sample size and power analysis*
Recruitment	15	Strategies for achieving adequate participant enrolment to reach target sample size<br>*see section Procedure, subsection recruitment*

Methods: Assignment of interventions (for controlled trials)

Allocation:

Sequence generation	16a	Method of generating the allocation sequence (eg, computer-generated random numbers), and list of any factors for stratification. To reduce predictability of a random sequence, details of any planned restriction (eg, blocking) should be provided in a separate document that is unavailable to those who enrol participants or assign interventions<br>*see section randomization*
Allocation concealment mechanism	16b	Mechanism of implementing the allocation sequence (eg, central telephone; sequentially numbered, opaque, sealed envelopes), describing any steps to conceal the sequence until interventions are assigned<br>*see section randomization*
Implementation	16c	Who will generate the allocation sequence, who will enrol participants, and who will assign participants to interventions<br>*enrolment: see section Quality Control, subheading Data Management and section Methods subsection Study design and Procedure, subheading Data collection*<br>*intervention assignment: see section Randomization*

Blinding (masking)	17a	Who will be blinded after assignment to interventions (eg, trial participants, care providers, outcome assessors, data analysts), and how<br>*Section Study Design*
	17b	If blinded, circumstances under which unblinding is permissible, and procedure for revealing a participant's allocated intervention during the trial<br>*n/a*

Methods: Data collection, management, and analysis

Data collection methods	18a	Plans for assessment and collection of outcome, baseline, and other trial data, including any related processes to promote data quality (eg, duplicate measurements, training of assessors) and a description of study instruments (eg, questionnaires, laboratory tests) along with their reliability and validity, if known. Reference to where data collection forms can be found, if not in the protocol<br>*see section Procedure, subheading Data collection, section Material (including Table 1) and section outcome measures*
	18b	Plans to promote participant retention and complete follow-up, including list of any outcome data to be collected for participants who discontinue or deviate from intervention protocols<br>*see section Procedure, subheading Participant retention and withdrawal*
Data management	19	Plans for data entry, coding, security, and storage, including any related processes to promote data quality (eg, double data entry; range checks for data values). Reference to where details of data management procedures can be found, if not in the protocol<br>*See section Quality Control, subsection Data Management, subheadings Data collection and Confidentiality, Data Access and Storage*
Statistical methods	20a	Statistical methods for analysing primary and secondary outcomes. Reference to where other details of the statistical analysis plan can be found, if not in the protocol<br>*See section Statistical Methods, subheading Statistical Analyses*
	20b	Methods for any additional analyses (eg, subgroup and adjusted analyses)<br>*n/a*
	20c	Definition of analysis population relating to protocol non-adherence (eg, as randomised analysis), and any statistical methods to handle missing data (eg, multiple imputation)<br>*See section Statistical Methods, subsection Statistical Analysis*

Methods: Monitoring

Data monitoring	21a	Composition of data monitoring committee (DMC); summary of its role and reporting structure; statement of whether it is independent from the sponsor and competing interests; and reference to where further details about its charter can be found, if not in the protocol. Alternatively, an explanation of why a DMC is not needed<br>*No serious safety concerns or unknown risks involved where adverse events would be expected*
	21b	Description of any interim analyses and stopping guidelines, including who will have access to these interim results and make the final decision to terminate the trial<br>*n/a*
Harms	22	Plans for collecting, assessing, reporting, and managing solicited and spontaneously reported adverse events and other unintended effects of trial interventions or trial conduct<br>*Section Quality Control, subheading Ethical considerations and Possible harm*
Auditing	23	Frequency and procedures for auditing trial conduct, if any, and whether the process will be independent from investigators and the sponsor<br>*See section Ethical considerations, subsection public dissemination, transfer and implementation*
**Ethics and dissemination**		
Research ethics approval	24	Plans for seeking research ethics committee/institutional review board (REC/IRB) approval<br>*See Sections Ethical considerations and Declarations, subheading Ethics approval and consent to participate*
Protocol amendments	25	Plans for communicating important protocol modifications (eg, changes to eligibility criteria, outcomes, analyses) to relevant parties (eg, investigators, REC/IRBs, trial participants, trial registries, journals, regulators)<br>*see sections Quality Control*
Consent or assent	26a	Who will obtain informed consent or assent from potential trial participants or authorised surrogates, and how (see Item 32)<br>*see sections Ethical considerations, subheading Informed Consent*
	26b	Additional consent provisions for collection and use of participant data and biological specimens in ancillary studies, if applicable<br>*n/a*
Confidentiality	27	How personal information about potential and enrolled participants will be collected, shared, and maintained in order to protect confidentiality before, during, and after the trial<br>*see sections Ethical considerations, subheading Confidentiality, Data Access and Storage*

Declaration of interests	28	Financial and other competing interests for principal investigators for the overall trial and each study site<br>*See section Declarations, subheading competing interests*
Access to data	29	Statement of who will have access to the final trial dataset, and disclosure of contractual agreements that limit such access for investigators<br>*see sections Ethical considerations, subheading Confidentiality, Data Access and Storage*
Ancillary and post-trial care	30	Provisions, if any, for ancillary and post-trial care, and for compensation to those who suffer harm from trial participation<br>*see section Ethical considerations, subheading Possible harm*
Dissemination policy	31a	Plans for investigators and sponsor to communicate trial results to participants, healthcare professionals, the public, and other relevant groups (eg, via publication, reporting in results databases, or other data sharing arrangements), including any publication restrictions<br>*see section Ethical considerations, subheading Public dissemination, transfer and implementation*
	31b	Authorship eligibility guidelines and any intended use of professional writers<br>*See section Ethical considerations, subheading Public dissemination, transfer and implementation*
	31c	Plans, if any, for granting public access to the full protocol, participant-level dataset, and statistical code<br>*See subsection Data management, subheading Confidentiality, Data Access and Storage*
**Appendices**		
Informed consent materials	32	Model consent form and other related documentation given to participants and authorised surrogates<br>*See section Declarations, subheading Availability of data and materials*
Biological specimens	33	Plans for collection, laboratory evaluation, and storage of biological specimens for genetic or molecular analysis in the current trial and for future use in ancillary studies, if applicable<br>*n/a*

*It is strongly recommended that this checklist be read in conjunction with the SPIRIT 2013 Explanation & Elaboration for important clarification on the items. Amendments to the protocol should be tracked and dated. The SPIRIT checklist is copyrighted by the SPIRIT Group under the Creative Commons "Attribution-NonCommercial-NoDerivs 3.0 Unported" license.
