# Supplementary material for: Project DECIDE II: evaluating the efficacy of supported advance care decision making within routine care in dementia: a randomized controlled trial
Source: BMC Med Ethics. 2025 Oct 8;26:124. doi: 10.1186/s12910-025-01290-6 (PMC12505856; doi:10.1186/s12910-025-01290-6)
Supplement: Supplementary file 3 — Supplementary Material 3. [file 12910_2025_1290_MOESM3_ESM.pdf]

Appendix

WHO Trial Data Registration Set

Category	Information
Primary registry and trial identifying number	drks.de<br>DRKS00036478
Date of registration in primary registry	17.04.2025
Source(s) of monetary or material support	German Federal Ministry of Research, Technology and Space (BMFTR)
Primary sponsor	Universität Siegen
Secondary sponsor(s)	-
Contact for public queries	*Julia Haberstroh, Universität Siegen,* 0049 (0)271 740-4053*, julia.haberstroh@uni-siegen.de*
Contact for scientific queries	*Julia Haberstroh, Universität Siegen,* 0049 (0)271 740-4053*, julia.haberstroh@uni-siegen.de*
Title	Project DECIDE-2 Evaluating the efficacy of supported Advance Care Decision Making within routine care in dementia: A randomized controlled trial
Countries of recruitment	Germany
Health condition(s) or problem(s) studied	Lack of effective supported decision-making strategies for people with dementia
Intervention(s)	Intervention 1: supported Advance Care Decision-Making within Dementia Care Management<br>Intervention 2: Dementia Care Management
Key inclusion and exclusion criteria	Inclusion criteria:<br>- confirmed diagnosis of mild to moderate dementia (F00-F03) or mild cognitive disorder (F06.7), diagnosed by a physician specialised in neurology or psychiatry and based on ICD-10 criteria.<br>Exclusion criteria:<br>- Severe dementia (MMSE score < 11)

Category	Information
	- lack of capacity to consent to medical research (and incapacity for supported decision-making with relative or proxy
Study type	Interventional allocation: randomised controlled study<br>Assignment: parallel group design<br>Masking: single-blind (participants are blinded)<br>Primary purpose: Increase the autonomy of PwD in making advance care and end-of-life decisions and reduce proxy burden
Date of first enrolment	Mai 2025
Target sample size	150
Recruitment status	Recruiting
Primary outcome(s)	- Prevalence and validity of advance healthcare planning documents<br>- Proxy burden
Key secondary outcomes	- Decisional conflict<br>- Patient autonomy<br>- Patient-caregiver congruence in preferences
